# Microfluidic Interrogation of Chitin-Induced Calcium Oscillations in the Moss *Physcomitrium patens*

**DOI:** 10.3390/plants15040582

**Published:** 2026-02-12

**Authors:** Vanessa Kamara, James Teague, Kathryn E. Pagano, Luis Vidali, Dirk R. Albrecht

**Affiliations:** 1 Department of Biomedical Engineering, Worcester Polytechnic Institute, Worcester, MA 01609, USA; vlkamara@wpi.edu (V.K.);; 2 Department of Mechanical Engineering, Worcester Polytechnic Institute, Worcester, MA 01609, USA; 3 Department of Biology and Biotechnology, Worcester Polytechnic Institute, Worcester, MA 01609, USAlvidali@wpi.edu (L.V.); 4 Bioinformatics and Computational Biology Program, Worcester Polytechnic Institute, Worcester, MA 01609, USA; 5 Department of Electrical and Computer Engineering, Worcester Polytechnic Institute, Worcester, MA 01609, USA

**Keywords:** calcium signaling, fungal predator, biotic stress, defense response, circadian rhythm

## Abstract

Plants defend against pathogens such as fungi by initiating coordinated structural and chemical responses. Pathogen perception triggers rapid cytosolic calcium influx and calcium oscillations that drive defense gene expression, yet the mechanisms by which these signals encode stressor intensity and propagate systematically remain unclear. Here, we present a microfluidic system to characterize intracellular calcium dynamics in protonemal colonies of the moss *Physcomitrium patens* (Hedw.) upon precise and reversible exposure to fungal chitin oligosaccharides. Epifluorescent imaging of cells expressing the calcium indicator GCaMP6f revealed a rapid, coordinated calcium response to chitin addition, followed by stereotyped oscillations that subsided quickly upon stimulus removal. We implemented an unbiased image segmentation algorithm using pixel-based *k*-means clustering to automatically locate regions with specific oscillatory signatures. Calcium dynamics were distinct across adjacent cells, distinguishable by cell type, and significantly modulated by circadian rhythm, adaptation time within the device, and stimulus timing. Cytosolic calcium oscillations, which rose and fell symmetrically within about 60 s, occurred spontaneously during the subjective night and following short adaptation periods. Chitin elicited strong oscillations with increased frequency, amplitude, and duration, and repeated pulses entrained regular, colony-wide oscillations at the stimulation interval. This study complements prior investigations of whole plant and growth tip dynamics and provides a quantitative framework to study calcium signaling in plants, including mechanisms of signal propagation and the role of oscillation frequency on gene expression.

## 1. Introduction

The ability of plants to sense and respond to biotic stressors, such as fungal, bacterial, and viral pathogens, is crucial for survival. Mosses share innate immune systems with vascular plants and evolved defense mechanisms that enable pathogen resistance [1]. For example, during the attack of a fungal predator, cells from the moss *Physcomitrium patens* (Hedw.) Mitt. detect pathogen-associated molecular patterns (PAMPs) at surface-localized pattern-recognition receptors (PRRs) to initiate an immune response [2]. Fungal PAMP perception is mediated in moss by the CERK1-LYK5 receptor complex, which is conserved across bryopyhtes, algae, and vascular plants [3]. Once activated, these cells exhibit a rapid influx of cytosolic calcium (Ca^2+^) [4] and simultaneous activation of the mitogen-activated protein kinase (MAPK) phosphorylation cascade [2]. This defense response propagates plant-wide, triggering reactive oxygen species (ROS) production, defense gene activation, and hormone synthesis, which ultimately lead to programmed cell death, growth inhibition, and cell wall reinforcement [5]. Many processes modulate plant–pathogen resistance, including circadian rhythms; for example, *Arabidopsis thaliana* susceptibility to the fungus *Botrytis cinerea* decreases at dawn, as plant defenses align with the rhythmic release of fungal spores [6].

Calcium signaling plays a vital role in these responses [7,8,9,10]. Stimulation of moss protonemal colonies with chitin oligosaccharides, a component of the fungal cell wall, causes colony-wide cytoplasmic Ca^2+^ oscillations and the expression of defense genes [4]. Calcium directly initiates this response, as Ca^2+^ influx alone by ionomycin treatment was sufficient to transiently elevate defense gene expression. These dynamic calcium signatures arise and propagate within and between cells though various ion channels, pumps, and calcium stores [11]; for example, Cyclic Nucleotide-Gated Channels (CNGCs) and Glutamate Receptor-Like channels (GLRs) mediate plasma membrane influx, while Two-Pore Channel 1 (TPC1) regulates vacuolar release [12]. Calcium levels and oscillation frequency then affect gene expression and other pathogen responses through numerous Ca^2+^-sensing molecules, including calmodulin (CaM), CaM-like proteins, Ca^2+^-dependent protein kinases (CDPKs) and calcineurin B-like proteins (CBLs), although the mechanistic details of calcium “coding” and decoding are largely undetermined [9,11,13].

This study addresses these questions using new experimental methods for the measurement and analysis of pathogen-triggered Ca^2+^ responses at colony-wide, single-cell, and subcellular levels. The moss *P. patens* is an advantageous model for studying plant–pathogen interactions due to its conserved defense mechanisms, genomic toolset, rapid growth, and small size [14]. In particular, the juvenile protonemal stage of *P. patens* has a filamentous morphology composed of linear and branching chains of chloronemal (CH) and caulonemal (CA) cells. Chloronemata contain an abundance of chloroplasts for photosynthetic energy production. Caulonemata are longer, thinner, and faster growing than chloronemata [15,16], attributes that are key for building plant mass and for nutrient acquisition. These cell types exhibit different calcium responses related to their position within the filament and growth status. For example, the apical regions of growing caulonema tip cells exhibit Ca^2+^ oscillations that promote the dissipation of actin filaments, as required for growth [4,17]. Prior studies utilizing protonemata have focused primarily on these growing tip cells, or on average calcium levels across the entire colony, which obscures cell type variation and signal propagation [18].

Widefield fluorescent imaging has been used to study calcium responses in vascular plants [19,20,21] and in moss [4,18] expressing various genetically encoded calcium indicators. Recently, microfluidic devices were fabricated to grow and image protonemal colonies while keeping them physically within a narrow imaging focal plane [16,22,23]. In addition, microfluidics enables the study of biological phenomena in precise chemical and mechanical microenvironments [24,25,26]. Specifically, laminar fluid flow permits precise and predictable spatiotemporal stimulus patterns, including the ability to rapidly introduce and remove chemicals [27]. Here, we present microfluidic devices capable of dynamically stimulating multiple protonemal colonies at once during timelapse calcium imaging. We demonstrate that protonemata housed in microfluidics trigger intracellular calcium waves comparable to studies that stimulated plants on agar coverslips. Further, we show for the first time how calcium dynamics react to stimulus removal and to repeated stimulation for different durations. We additionally examined the effects of adaptation time within the microfluidic environment, the timing of stimulation relative to circadian cycles, and variations among cell types.

In functional imaging studies, data analysis can be a substantial bottleneck [28]. Each timelapse image stack may require manual annotation and corrections to address sample movement or illumination artifacts. To increase experimental throughput, reduce bias, and increase reproducibility, we developed and validated a semi-automatic, unbiased clustering algorithm to segment contiguous regions within the protonemal colonies by their calcium dynamics. These regions included whole individual cells as well as subcellular regions. We report the spontaneous and chitin-elicited responses across cell types and subcellular regions to long and short chitin exposures, and highlight differences in response due to time of day and adaptation in the device. Together, these microscopy, microfluidic, and data analysis methods enable the broad study of protonemal responses to dynamic, reversible stimuli and the underlying calcium signaling patterns.

## 2. Results

### 2.1. Microfluidic Stimulation and Calcium Recording

**Figure 1:**
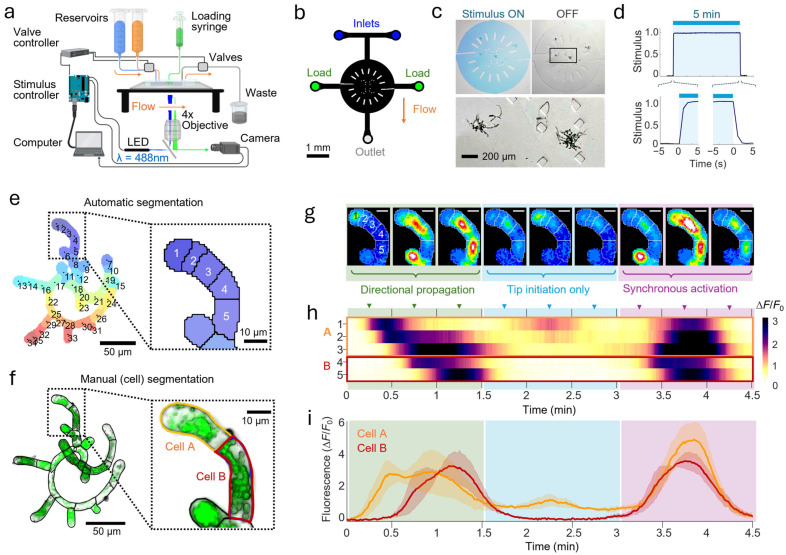
Microfluidic system for protonemal stimulation and calcium imaging. (**a**) Diagram of image acquisition setup. LED, light-emitting diode. (**b**) Diagram of the microfluidic device and flow path. Fluid flows from one of two reservoirs (stimulus or moss medium) via inlets (blue) to the outlet. Colonies are loaded from either of two side ports (green). (**c**) Fluid in the arena can be switched rapidly and reversibly between the stimulus (blue) and moss medium (clear) solutions, exposing multiple colonies to different chemical environments (inset). Here, blue dye was used to visualize the stimulus solution. (**d**) Switching between solutions is rapid and repeatable. Dye intensity plots show the mean ± SEM of five repeated pulses, each 5 min in duration. (**e**) An automated algorithm generates regions of interest (ROIs) by clustering pixels with similar fluorescence dynamics (see Materials and Methods). ROIs represent cells or subcellular regions. (**f**) Brightfield image of the same plant as in (**e**), manually segmented along cell wall boundaries. Typical cells are represented by 1–3 ROIs: cell A (orange) is represented by ROIs 1–3 and cell B (red) by ROIs 4,5. The green fluorescence overlay represents cell wall staining using Direct Yellow 96 and chloroplast autofluorescence. (**g**) Timelapse frames (30 s interval) depicting calcium fluctuations in the five automatically generated ROIs underlying cells A and B. Scale bars = 10 μm. (**h**) Heatmaps of the ROI-averaged fluorescence values which clearly demonstrate different calcium wave behaviors: (1) propagating from the apical tip in the basal direction (green), (2) tip initiation without propagation (blue), and (3) synchronous activation of all ROIs and dissipation. (**i**) Averages for cells A and B show similar patterns, but obscure subcellular propagation, seen as increased SD amplitude (shading).

Protonemal colonies expressing the cytoplasmic calcium indicator GCaMP6f [4,29] were imaged in a microfluidic flow chamber to visualize spontaneous and chitin-induced Ca^2+^ responses from multiple colonies at once (Figure 1a,b). Colonies between 6 and 8 days old were loaded into the fluid-filled microfluidic channels by syringe (Figure 1b,c) and allowed to adapt to the microfluidic environment for up to 3 h in darkness with a continuous flow of moss medium (described in Methods Section 4.2). Calcium levels were recorded at 1 fps by epifluorescence microscopy. The application and removal of stimulus solutions was automated and reversible, switching completely within 2 s and remaining stable for minutes to hours (Figure 1c,d).

### 2.2. Identification of Protonemal Regions and Calcium Waves

Image stacks were cropped and segmented to automatically identify regions of interest (ROIs), as described in detail in Methods Section 4.8 (Figure 1e). Briefly, *k*-means clustering grouped individual image pixels by similarity of their fluorescence intensity dynamics, treating each time point as a separate dimension, followed by editing for ROI connectivity and size limits (see Materials and Methods (Section 4), below). To assess automatic segmentation quality, protonemal cell borders were stained after calcium recordings using Direct Yellow 96 and manually segmented (Figure 1f). Typical cells were represented by one to three ROIs, reflecting distinct subcellular calcium dynamics.

Observation of tip cells revealed three common calcium oscillation patterns (Figure 1g, Supplementary Video S1). First, the apical tip region could initiate a calcium wave, followed by directional propagation to subapical cells over about 1 min. Second, the tip initiation could subside without any wave or propagation to adjacent subcellular regions or cells. Third, synchronous activation of cells and regions could arise and subside without directional propagation (Figure 1h). Because directional propagation often occurred within protonemal cells, averaging fluorescent intensity within cell regions could obscure these dynamics (Figure 1i). Therefore, we used the automatically segmented, subcellular ROIs to investigate colony-wide responses to different chitin stimulation patterns and other experimental conditions.

### 2.3. Chitin Elicits Distinct Calcium Oscillations Across Protonemal Colonies

**Figure 2:**
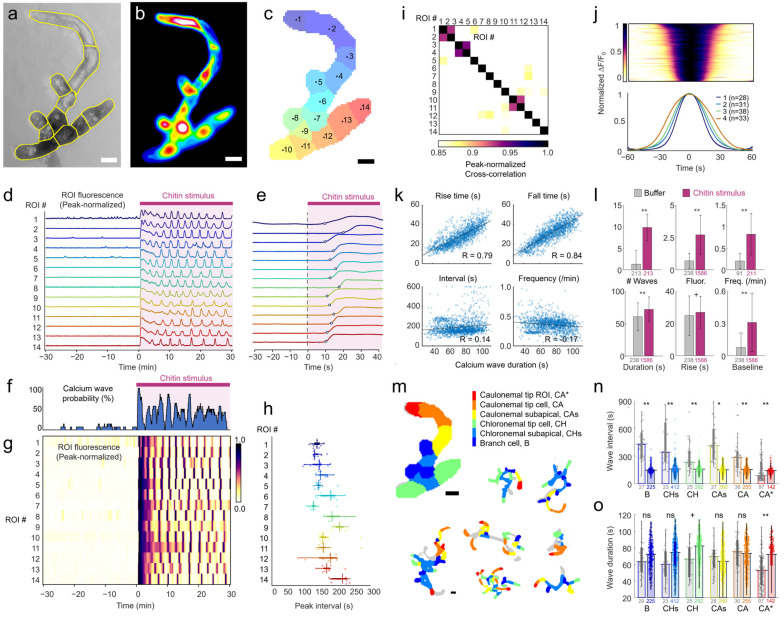
Chitin elicits persistent colony-wide calcium oscillations. (**a**) Example moss protonemal colony in brightfield imaging with cell borders delineated in yellow. (**b**) Fluorescent image of GCaMP indicating intracellular calcium levels, taken from one frame of a 60 min timelapse. (**c**) Automatically segmented ROIs. Scale bars (**a**–**c**), 25 µm. (**d**) Calcium fluorescence integrated across each ROI, normalized to the peak response at chitin stimulus onset. Colonies in the microfluidic device were allowed to adapt for 3 h, then imaged for 60 min with 30 min of buffer and 30 min of 2.2 mg/mL chitin stimulus. (**e**) Expanded timescale of calcium response in (**d**), showing response latency (stimulus onset to 10% peak intensity). (**f**) The probability of calcium waves rose at chitin onset, followed by oscillating probability during continuous 30 min stimulation. (**g**) Heatmap of normalized calcium fluorescence from (**d**). (**h**) Intervals between calcium waves varied across ROIs. (**i**) Maximum cross-correlation heatmap shows little temporal correlation among ROIs, except for ROIs within the same cell (1–2, 3–4, 10–11). (**j**) Individual normalized calcium waves (*n* = 133 waves) show symmetric onset and offset within a tight duration range, reported as full width spanning 10% of peak prominence (FW10). Quartile averages are plotted below. (**k**) Calcium wave duration was strongly and positively correlated with wave rise and fall times (10% to 90%) but only weakly correlated with wave interval and frequency. (**l**) Comparison of calcium dynamics before (gray) and after (purple) chitin stimulation for nine colonies. Chitin increased the number of waves per 30 min, the peak calcium fluorescence, the wave frequency, and the baseline calcium signal (relative to wave peak height). Chitin increased the duration of each calcium wave (FW10) and, to a lesser extent, the rise time (10% to 90%). (**m**) ROIs were mapped to six moss ROI types and regions, as indicated for the colony in (**a**–**e**) and seven additional colonies. Caulonema tip cells were divided into the apex tip region (CA*) and the remainder of the cell (CA). Both CA and chloronema (CH) tip cells were distinguished from adjacent subapical cells (CAs and CHs) and branch (B) cells budding from central branch points. (**n**) Across eight colonies, chitin stimulation elicited faster calcium waves in all ROI types except CA*, whose interval increased. Values were log-transformed before one-way ANOVA. Bars represent median, interquartile range, and individual wave data points (number listed at the base of each bar). (**o**) Calcium wave durations were dependent on cell type and region, plotted as in (**g**). Differences between groups were analyzed via one-way ANOVA. **, *p* < 0.0001; *, *p* < 0.001; +, *p* < 0.01; ns, not significant.

In the first experiment set, colonies were allowed 3 h to adapt to the microfluidic arena in darkness after entering the device. Each experiment included a 30 min period exposing colonies to moss media without stimulus, followed by a 30 min period of chitin exposure (Figure 2). A small, representative 11-cell colony is shown in Figure 2a, divided into 14 ROIs with distinct calcium dynamics (Figure 2b,c). Calcium fluorescence remained low during the 30 min medium flow, followed by a rapid rise upon chitin addition that subsided over 3 to 7 min (see Supplementary Video S2). During the initial response period, some cells exhibited a burst of peaks before they transitioned to regular, repeating calcium waves (Figure 2d). The initial response latency between chitin addition and calcium rise varied from 16 to 32 s across the colony (22.6 ± 4.5 s, mean ± SD to 10% peak level, Figure 2e). The instantaneous probability of calcium waves across all 14 ROIs was low during buffer (5.2 ± 4.7%), then reached 100% within 1 min of stimulus onset (Figure 2f), and settled to an average of 44% at any moment during chitin stimulation.

We identified calcium oscillations by fluorescence peaks, then analyzed and quantified their magnitude and timing, including rise time, fall time, duration, and interval. Calcium fluorescence intensity varied across ROIs (see Methods and Figure 6). However, when normalized to the peak initial chitin response, calcium wave dynamics appeared similar both within and across ROIs (Figure 2d). Calcium oscillations occurred at different intervals, ranging from 1.1 to 4.6 min (63–276 s, Figure 2g,h). Within each ROI, interval variance was lower (CV 19.2 ± 11.1%) than across all ROIs (CV 45.4%), indicating that each cell had a characteristic oscillation frequency (Figure 2h). Adjacent ROIs did not oscillate in synchrony, as might be anticipated from the diffusion of calcium across plasmodesmata. Instead, only ROIs within the same cell showed high response cross-correlation, a measure of synchrony independent of fixed phase shifts (Figure 2i). Notably, the site of wave initiation and the direction of propagation were not static throughout stimulation, even within a single cell.

We next asked whether individual Ca^2+^ wave dynamics (for example, rise and fall times) were stereotyped or scaled with overall oscillation frequency. Calcium wave duration ranged from 30 to 110 s and was only slightly correlated with wave interval (R = +0.14) and its inverse, wave frequency (R = –0.16) (Figure 2j). In contrast, rise and fall times correlated strongly and equally with wave duration (R = 0.79–0.84), reflecting a symmetric, Gaussian wave shape (Figure 2k). Together, these results suggest that calcium waves are initiated in different cells at different intervals, but individual wave dynamics are similar and preserve the bell curve shape as calcium levels rise and fall.

These results were representative across nine protonemal colonies divided into 213 ROIs. Before chitin addition, spontaneous calcium waves were variable and rare (1.3 ± 3.2 per 30 min), increasing dramatically and consistently across ROIs during chitin exposure (9.6 ± 3.1 waves per 30 min). Chitin-induced oscillations were four times stronger (ΔF/F_0_ = 2.7 vs. 0.7, *p* < 0.0001) than spontaneous Ca^2+^ oscillations and occurred four times more often (Figure 2l). Calcium waves in chitin occurred at an average frequency of 0.8 ± 0.5 min^−1^ (interval 159.7 ± 64.5 s, interquartile range 127.5 to 181.0 s) in the microfluidic setup, comparable to prior observations [4]. Individual colonies responded similarly, with colony-wide mean chitin-induced wave intervals ranging from 142 to 218 s (Supplementary Figure S1). Chitin-induced waves were ~20% longer in duration (72 s vs. 61 s) than spontaneous waves, exhibited slightly longer rise times (27 s vs. 25 s), and coincided with higher baseline calcium levels preceding and following each oscillation.

### 2.4. Calcium Wave Dynamics Differ Among Cell Types and Subcellular Domains

We next assessed whether different cell types within the protonemal filaments exhibited distinct calcium dynamics, propagation rates, or directionality. In developing moss colonies, chloronemal cells dominate the early regenerative phase before spatial cues and auxin signaling drive the differentiation of the apical cells into caulonemata [16]. These growing apical caulonemal cells exhibit a tipward Ca^2+^ gradient with rapid oscillations (<1 min interval), a hallmark of tip-growing plant cells [17]. While it is known that chitin perception rapidly inhibits growth and dissipates this apical gradient within 1–2 min [2,4], a systematic characterization of Ca^2+^ dynamics in subapical cell types had not yet been performed.

The automatic segmentation algorithm extracted 1 to 3 ROIs per cell. In particular, caulonemal (CA) tip cells were often separated into multiple ROIs, capturing the distinct dynamics exhibited near the apical tip (CA*) prior to chitin application. Spontaneous calcium waves were rare in most cell types except the CA* apical tip region, which exhibited waves with a median interval of 85.5 s (59.0–235.8 s interquartile range), consistent with prior reports of moss cultured on cover slips. Chitin addition induced similar calcium waves across the whole protonemal colony, oscillating at a mean interval of 156.4 ± 6.7 s across all cell types (Figure 2n). The fast spontaneous calcium waves in the CA* apical tip regions slowed 39% by chitin exposure, but still oscillated slightly quicker (144.6 s interval) than both chloronemal subapical cells (CHs) and tip (CH) cells (167.7 s and 161.8 s, respectively, *p* < 0.05). Additionally, chitin increased wave duration in both CA* apical tip regions (+26%, *p* < 0.0001) and CH cells (+19%, *p* < 0.001). No other cell types featured a significant change in wave durations when compared to their baseline values (Figure 2o). Within cell types, subapical cells exhibited ~5 s shorter duration waves than tip cells during chitin stimulation, for both chloronemal (CHs 72.4 s vs. CH 79.1 s) and caulonemal (CAs 66.2 s vs. CA 71.4 s, CA* 70.1 s) cells, while branching cell wave durations were similar to their neighboring subapical cell (B 71.8 s).

### 2.5. Device Adaptation Time

**Figure 3:**
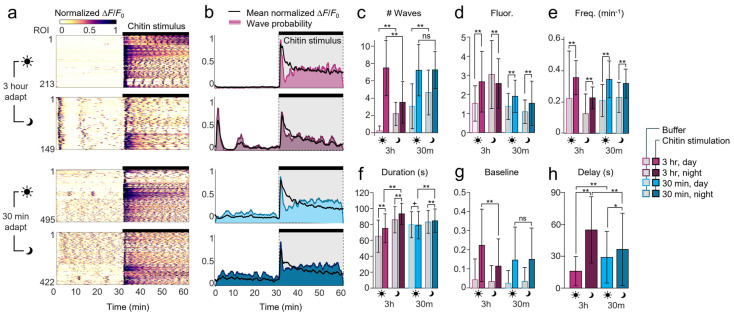
Calcium oscillations are modulated by adaptation time and by circadian rhythms. (**a**) Normalized heatmaps of colony ROIs during 30 min buffer flow and 30 min chitin stimulation. Protonemal colonies were adapted in the microfluidic device for 3 h or 30 min, and images were taken during daytime (dawn-to-dusk) or nighttime, as indicated by the sun and moon icons. Calcium fluorescence is normalized to the minimum and peak intensity per ROI. Data represent *n* = 149–495 ROIs from 3 to 9 plants. (**b**) Mean ROI responses are shown as normalized fluorescence intensity averaged across all ROIs per condition (black line) or as the probability of a calcium wave over time (shading). Bars and shading represent the chitin stimulation time (from 30 to 60 min). (**c**–**g**) Comparison of calcium wave dynamics before and after chitin addition across adaptation and day/night conditions. Data represent: (**c**) the number of waves per 30 min period (buffer or stimulus); (**d**) peak calcium wave fluorescence change relative to baseline, ΔF/F_0_; (**e**) calcium wave frequency per minute; (**f**) calcium wave duration (above 10% of maximum prominence, FW10); and (**g**) relative calcium fluorescence baseline at the beginning of each wave. **, *p* < 0.0001; *, *p* < 0.001 for buffer vs. stimulus or specified comparison; +, *p* < 0.0001 compared with buffer, 3 h adaptation, daytime condition. Data represent mean and SD, *n* = 91–4592 waves. (**h**) The response delay (s) between stimulus application and initial response onset (above 10% of maximum prominence). **, *p* < 0.0001; *, *p* < 0.001 compared between both time of day and adaptation time duration. Data represent *n* = 149–407 waves.

Protonemal colonies introduced into the microfluidic arenas experience a different local environment compared to cultivation. The gentle injection into the device may also cause brief mechanical perturbation upon loading, and slow microfluidic flow imposes a weak, constitutive shear stress. We therefore tested how adaptation time in the device influenced chitin-induced calcium responses (Figure 3a,b). Colonies allowed to acclimate to darkness and the microfluidic environment for only 30 min before imaging showed several prominent spontaneous calcium waves (3.1 ± 2.6 per 30 min) before the addition of chitin, unlike colonies acclimated for 3 h (0.2 ± 0.6 per 30 min, Figure 3c). After stimulation, no difference was noted in the number, frequency, or duration of calcium oscillations, although peak and baseline GCaMP fluorescence levels were reduced with short 30 min adaptation (Figure 3c–g). Thus, the extended 3 h adaptation time quieted spontaneous calcium waves and magnify chitin-triggered responses, with no change in stimulated wave dynamics.

### 2.6. Circadian Modulation of Ca^2+^ Waves

Plants modulate core processes such as defense and growth according to the time of day, due to their reliance on light for energy and its diurnal variation [30,31]. Moss protonemal cells, like vascular plants, exhibit a Circadian Clock-Associated 1 (CCA1) homolog whose expression becomes maximized near dawn [32]. We therefore investigated whether chitin-induced calcium oscillations were modulated by stimulation time relative to the day/night circadian cycle.

Whereas long-adapted colonies examined during daytime showed little spontaneous activity prior to chitin stimulation (Figure 3a), strong calcium waves appeared consistently in nighttime colonies within minutes of timelapse acquisition (89% of ROIs active at 85 s, Figure 3b). These nighttime responses occurred shortly after the first exposure of blue GCaMP excitation light. Following about 10 min of quiescence, about 90% of ROIs displayed additional spontaneous Ca^2+^ waves for the remainder of the baseline period. During this time, Ca^2+^ waves traveled slowly within and between cells (Supplementary Video S3). Thus, colonies were sensitive to blue light stimulation, but only at night and after long adaptation in darkness, consistent with prior reports [33].

After chitin stimulation, long-adapted colonies exhibited nearly twice as many calcium waves in daytime (7.5 ± 3.2 waves) than in nighttime (3.5 ± 2.4 waves, *p* < 0.001), while no difference was seen for the short-adapted group (7.2 ± 3.0 vs. 7.2 ± 2.1 waves, Figure 3c). The peak fluorescence values increased significantly during stimulation for all experimental groups (Figure 3d), excluding the blue-light response in long-adapted nighttime-stimulated colonies. Spontaneous wave frequency was lower in nighttime long-adapted colonies than daytime, but equal in short-adapted colonies (Figure 3e). Wave frequency increased in all conditions compared with spontaneous waves, and faster oscillations were similar across colonies, regions, and cell types (Supplementary Figure S2). Calcium waves were ~10 s longer during stimulation, and ~20 s longer at night than during the day in long-adapted colonies, but similar for all conditions in short-adapted colonies (Figure 3f). Baseline calcium levels during stimulation were modulated by time of day (lower at night), but only in long-adapted colonies (Figure 3g).

In spontaneously active regions of long-adapted colonies at night, the initial chitin-induced response was delayed three-fold (55 ± 32 s) compared with the daytime (16 ± 14 s, *p* < 0.001), suggesting that recent calcium activity slowed subsequent stimulus-evoked responses in long-adapted colonies (Figure 3h). In short-adapted colonies, the response delays were more similar but still longer at night (37 ± 34 s night vs. 29 ± 24 s day, *p* < 0.01). The initial chitin-onset response was more synchronized in daytime-stimulated colonies (especially after long adaptation, 14 s SD) and more variable in nighttime (32–34 s SD).

Taken together, the long 3 h adaptation time in the microfluidic environment decreased baseline calcium levels and spontanous oscillations, but did not affect chitin-stimulated activity. Circadian rhythms affected spontaneous activity, blue light sensitivity, and stimulated wave frequency after long adaptation, but had little effect in short-adapted colonies, suggesting that the experimental microenvironment plays a stronger role in calcium dynamics.

### 2.7. Calcium Waves Elicited by Repeated Stimuli

A key benefit of microfluidic stimulation is the ability to deliver repeated pulses separated by complete wash-out of the stimulus solution. To analyze the repeatability of chitin-elicited calcium signals, we applied five consecutive 5 min stimulus pulses, with 5 min of buffer flow between each stimulation. A similar duration exposure to chitin oligosaccharides was sufficient to upregulate defense gene expression [4,16].

**Figure 4:**
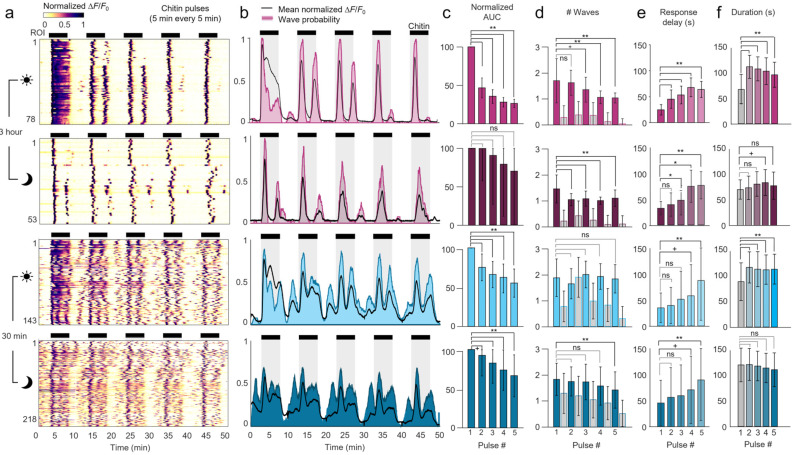
Repeated chitin pulses elicit repeated calcium waves. (**a**) Normalized heatmaps of protonemal colony ROIs during five 5 min chitin stimulation pulses. Colonies were adapted in the microfluidic device for 3 h or 30 min and imaged during daytime (dawn-to-dusk) or nighttime, as indicated. Calcium fluorescence is shown normalized to the minimum and peak intensity per ROI. (**b**) Mean ROI responses are shown as normalized fluorescence intensity (black line) or as instantaneous probability of a calcium wave over time (shading). Black bars and gray shading represent chitin addition. (**c**–**f**) Comparison of calcium wave dynamics throughout the 5 stimulus additions. Data represent (**c**) the normalized area under the curve (AUC) of each stimulus repetition normalized to the response to the first stimulus pulse; (**d**) the average number of waves exhibited per ROI per 5 min time period; (**e**) the response latency between the five chitin additions and the initiation of the first chitin-induced wave (above 10% of maximum prominence); and (**f**) calcium wave duration (above 10% of maximum prominence, i.e., FW10). **, *p* < 0.0001; *, *p* < 0.001 for buffer vs. stimulus or specified comparison; +, *p* < 0.0001 compared with buffer, 3 h adapt, daytime condition. Data represent mean and SD, *n* = 53–218 waves from 2 to 5 colonies.

Calcium waves were initiated by each individual chitin application and ceased after each chitin removal, with dynamics that were modulated by adaptation time and by time of day (Figure 4a,b). The long-adapted protonemal colonies featured little calcium activity in moss medium between pulses, like the quiet pre-stimulation period in Figure 3. In the daytime, the first pulse produced a sustained calcium response, similar to the first few minutes of the 30 min stimulation. In contrast, later pulses produced calcium waves that were highly synchronized, with nearly all ROIs initiating calcium waves about 1 min after stimulation (Figure 4b, Supplementary Video S4). Responses diminished with repeated chitin pulses, as integrated fluorescence decreased steadily over time (Figure 4c). Relatedly, the 5 min chitin pulses produced either one or two synchronized calcium waves, with the number of doubled waves decreasing for later repeats as wave interval increased (Figure 4d).

Calcium waves also became more delayed and less synchronized for each chitin pulse. Response delay between stimulus application and response initiation increased three-fold from 25 s to 64 s over five pulses (Figure 4e). Response synchrony decreased over time, as seen by an increase in the standard deviation of delay time from 10 to 16 s (Figure 4e). Wave duration also decreased over repeated pulses, except the first pulse wave appeared shorter as calcium levels remained above baseline (Figure 4f).

At nighttime, the long-adapted colonies showed similar trends in response adaptation to daytime. Each 5 min chitin pulse elicited one and occasionally two calcium waves, but less frequently than during daytime, as wave intervals were slightly longer at night (Figure 4d). At nighttime, waves had 20–30% longer response delays than during daytime, increasing from 33 s to 76 s from the first to the fifth pulse. Nighttime waves also showed 30 to 60% less response synchrony than during daytime, as delay standard deviation rose to 13 s to 26 s from pulse 1 to 5 (Figure 4e). Wave durations were more consistent across repeats at night (Figure 4f).

Short-adapted colonies had more spontaneous calcium waves outside of chitin stimulation (about one per 5 min) and more chitin-evoked waves (about two per 5 min cycle) than long-adapted plants (Figure 4a,d). While the number of waves per pulse remained steady across repeats, response adaptation manifested as a steady decline in integrated response over time (Figure 4c). Response delays were elevated with short adaptation time, with a mean delay increasing from 35 to 87 s over the five repeats during the day, and from 45 to 90 s at night (Figure 4e). Response synchrony was substantially lower in short-adapted colonies. Again, time of day played little role in short-adapted colonies, with similar calcium wave number, magnitude, onset delay, or duration in day and night experiments.

### 2.8. Protonemal Colony-Wide Calcium Oscillations Driven by Short-Pulse Stimulation Frequency

**Figure 5:**
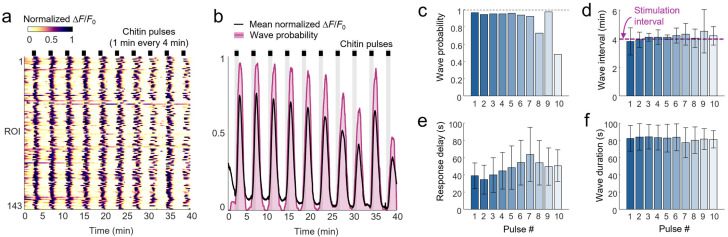
Calcium wave frequency is driven by chitin stimulation frequency. (**a**) Normalized heatmap of protonemal colony ROIs during ten 1 min chitin stimulation pulses every 4 min. The colony was adapted in the microfluidic device for 1 h before imaging. Calcium fluorescence change (ΔF) is normalized to the minimum and peak intensity per ROI. Data represent *n* = 143 ROIs from one colony. (**b**) Mean ROI response shown as normalized fluorescence intensity averaged across all ROIs per condition (black line) or as probability of a calcium wave over time (shading). Bars and shading represent chitin pulses. (**c**–**f**) Comparison of calcium wave dynamics across 10 repeated stimulus pulses, including (**c**) the probability of eliciting a calcium wave; (**d**) intervals between calcium waves; (**e**) response delay between chitin addition and calcium fluorescence rise (to 10% of maximum); and (**f**) calcium wave duration. Data represent mean and SD, *n* = 71–146 waves per pulse.

Since five-minute duration pulses elicited an initial calcium wave, followed occasionally by a second wave, we reasoned that shorter chitin stimulation should elicit a single, synchronized calcium wave across the colony for each brief chitin pulse. Indeed, 1 min of chitin exposure was sufficient to elicit a calcium wave in nearly every ROI (Figure 5a–c, Supplementary Video S5). Over 88% of all ROIs responded to all ten repeated 1 min pulses, and 95% of them initiated calcium waves to the first seven pulses (Figure 5c). Calcium oscillations were effectively driven at chitin stimulation frequency, with a median interval of 244 s (4.07 min), or just 2% above the 4 min chitin pulse interval, and were consistent across the ten repeats (Figure 5d). Calcium waves were initiated 47.5 ± 24 s (SD) after chitin addition, with a gradual increase in response latency from the first pulse (39.0 ± 14.8 s) to the last four pulses (56.1 ± 25.7 s) (Figure 5e).

Wave durations averaged 82.1 ± 15.0 s and were consistent for all ten pulses (Figure 5f) and similar to durations for 30 min stimulations (Figure 3f). Chitin removal did not suppress or shorten calcium wave activity; in fact, nearly all (99.8%) waves concluded at least 20 s *after* chitin was removed (mean 67.8 ± 24.1 s). About 26% of waves were initiated after chitin removal (Supplementary Figure S3). This suggests that the process of wave initiation begins before calcium rises to detectable levels and is not terminated once stimulation ceases.

## 3. Discussion

This study expands upon prior investigations of calcium oscillations in *P. patens* moss protonemal cells, combining epifluorescent calcium imaging of protonemal colonies expressing the GCaMP indicator with microfluidic devices for rapid and reversible chemical stimulation. We demonstrated tip-localized calcium oscillations indicative of tip growth and colony-wide calcium oscillations upon introduction of chitin oligosaccharides, and measured calcium dynamics that resembled prior studies on agar and in microfluidic growth chambers [4,17]. We further illustrate, for the first time: (1) calcium response variations among different cell types and subcellular regions; (2) response modulation by adaptation time and by experimental time of day relative to circadian rhythms; (3) responses to the sudden *removal* of chitin stimulus; (4) responses to repeated pulses and response adaptation over repeated stimulation; and (5) the ability to drive colony-wide calcium oscillations at a prescribed frequency. We characterized calcium wave dynamics for each experimental condition across several parameters, including rates, durations, intervals, probabilities, and variabilities, as a baseline for comparison and to inform future experimental designs. We validated an unbiased, pixel-based *k*-means clustering approach to automatically extract ROIs exhibiting calcium wave dynamics which differ in time of initiation, direction of propagation, or oscillation frequency, and developed data analysis software to extract, summarize, and combine results from multiple individual colonies.

The automatic segmentation algorithm reduced data analysis time 10-fold, from upwards of an hour (for manual segmentation of a typical-sized week-old protonemal colony) to under 5 min, and identified physiologically relevant subcellular regions that exhibited unique calcium dynamics. In particular, the apical region of the caulonemal tip cell (CA*) was automatically distinguished from the rest of the cell (for example, ROI #1 in Figure 2), due to rapid calcium fluctuations prior to chitin addition, characteristic of tip growth. While newer machine learning methods based on matrix factorization and convolutional neural networks have been developed for the segmentation of plant cells [34,35] and neurons [36], these methods are more computationally intensive and beneficial for handling fluctuating background signals and overlapping cells. Here, our thin microfluidic arena minimized background fluctuations and cell overlaps, allowing the use of a simple, effective, and efficient clustering algorithm focused directly on differences in calcium dynamics. This segmentation method requires a change in calcium fluorescence during each recording, which we achieved by chitin stimulation, although a few protonemal cells that remained inactive or constitutively bright were excluded from analysis. The potential downsides of pixel-based *k*-means clustering, namely requiring an initial cluster number (*k*) and ignoring spatial connectivity, were effectively mitigated by post-processing: grouping ROIs by pixel connectivity, splitting noncontiguous ROIs, and pruning sub-minimum-sized regions representing noise.

This study also validated the use of microfluidic devices for studying chitin-induced Ca^2+^ waves at the protonemal colony, cell, and subcellular levels. The prolonged 30 min chitin stimulation experiments (Figure 2 and Figure 3) most closely matched prior studies exposing moss protonemal tissues on agar pads to a single, sudden increase in chitin. Here, moss housed in microfluidic arenas demonstrated quantitatively similar dynamics to protonemata on agar, with a similar rapid increase in cytosolic Ca^2+^ and comparable colony-wide oscillation intervals (2–3 min interquartile range, vs. over 2 min interval on agar [4]). Before stimulation, growing apical regions of the caulonemal cells (CA* region) oscillated at a rate comparable to agar (2.4–2.6 per min vs. ~2 per min [4]). We further characterized cell type differences and found that, while the frequency of chitin-induced waves was relatively consistent across all cell types, the waves in chloronemal and branch cells were slower and longer than those in caulonemal cells. Chloronemal tip cells (CH) exhibited Ca^2+^ waves and chitin-induced changes uniformly across the cell, with similar dynamics to those seen in the apical subcellular region of caulonemal tip cells (CA*), reflective of growth occurring in both tip cell types but more apically localized in the polarized caulonemal cells [15].

Chitin stimulation induced stereotyped, high-amplitude calcium oscillations with a symmetrical rise and fall that scaled with wave duration, which varied four-fold across all conditions from about 30 to 120 s (mean 77 s; median 79 s; 65–91 s interquartile range). These dynamics did not correlate with oscillation interval, suggesting that wave initiation occurred by signaling processes separate from calcium levels alone. Oscillation intervals were consistent within each cell and ROI, at least over the time scale of an hour. Chitin-induced waves were initiated shortly after stimulus presentation, with an onset delay that varied several-fold (mean 32 s; median 24 s; 14–40 s interquartile range). These delays align with the timing of CERK1 receptor activation by chitin and downstream MAPK phosphorylation [2]. Interestingly, initiation delays were longer after recent calcium activity, and shortest after quiescent periods, suggesting a “refractory period” in which components involved in calcium wave initiation inactivate or become sequestered during calcium activity and slowly recover responsiveness. Initiated waves followed their prescribed rise and fall and could not be terminated by removal of the stimulus, again suggesting a disconnect between the initiation and the progression of each calcium oscillation. Like other “all-or-none” excitable systems, such as neural action potentials, these oscillations appear to have an internal initiation threshold, a standardized, maximal, nongraded response, and a recovery period before “firing” again.

In vascular plants, cytosolic calcium spikes are typically asymmetric, rising rapidly through the activation of Ca^2+^ influx channels and decaying slowly via pumps such as auto-inhibited Ca^2+^-ATPases (ACAs) [11,37]. Similarly, the initial moss response to chitin onset was a rapid calcium rise followed by a slow baseline decay (see Figure 3a,b). However, the symmetry of subsequent oscillations suggests the involvement of distinct channels and transporters from this initial response. For example, the high density of moss ACA1/2 in the subapical plasma membrane might act to quickly counteract influx and accelerate a return to baseline [38]. Although the measured oscillation symmetry in our experiments could be affected by calcium sensor binding kinetics, GCaMP6f has a fast off-rate [29], suggesting a genuine balance between calcium influx and efflux during oscillations.

Circadian rhythms enable plants to manage daily cycles such as the optimization of photosynthesis, stomatal opening, and metabolic processes, to align with diurnal light and dark cycles, boost growth, and enhance fitness [31]. The responsiveness of cells to external stimuli and internal signals is regulated by circadian rhythms through differential gene expression, alterations in membrane voltage, and post-translational modification [39]. The circadian clock in *P. patens* is simpler than in vascular plants, but retains homologs of several key regulators. For example, cryptochrome CRY1 encodes when blue light affects gene expression by translocation into the nucleus, and clock genes such as CCA1 determine how strongly blue light is perceived by modulating blue-light receptors PHOT1/2, which implement phototropic responses at the cellular level [40]. Moss exhibits Ca^2+^ transients shortly after blue light exposure [33], and we observed that blue light excitation of the calcium sensor could directly trigger calcium responses under specific conditions (long adaptation, subjective nighttime), with similar duration and periodicity to prior studies [41].

Light sensitivity and circadian regulation motivated the comparison of calcium activity after different pre-experiment adaptation times and at various times during the subjective day and night. We found that the time of day affected spontaneous calcium dynamics prior to chitin stimulation in long-adapted protonemal colonies: in daytime, only caulonemal apical tip regions exhibited fluctuations in Ca^2+^ indicative of tip growth, while the rest of the cells remained silent. In contrast, colonies stimulated at subjective night exhibited the aforementioned blue-light response during the first minute of imaging, followed by spontaneous calcium waves that propagated within and between adjacent cells, perhaps through plasmodesmata or apoplastic mechanisms [42]. Unlike long-adapted colonies, short-adapted colonies responded similarly at different times of day and did not show a coordinated blue-light response, suggesting that prolonged adaptation in the microfluidic device is required for sensitivity to circadian rhythms and light. Short-adapted colonies also exhibited far more spontaneous Ca^2+^ activity prior to chitin stimulation, regardless of time of day, likely a residual response to the novel microfluidic environment.

Repetitive stimulation of living systems often elicits diminishing responses over time, such as the adaptation of internal signals and the habituation of organism behavior. A related memory phenomenon, priming, refers to the exogenous application of a substance to plants to enhance their natural defense responses and their tolerance to future stress presentations [43]. Because the microfluidic system can reversibly apply and fully remove chemical stimuli, we assessed, for the first time, whether calcium responses adapt or change over multiple, identical chitin pulses. Indeed, stimulation with five cycles of 5 min chitin and 5 min of rest revealed a gradual decline in response magnitude and wave probability, with progressively lower calcium response levels, fewer calcium waves, longer wave intervals, longer onset delays, and decreased wave synchronicity (Figure 4). While the exact mechanisms of desensitization in moss colonies are currently unclear, evidence from *A. thaliana* suggests that CERK/LYK5 receptor endocytosis [3,44] or ubiquitination [45] could desensitize chitin perception. In addition, the regulation of calcium release channels could play a role in the downstream calcium response. In particular, the vacuolar cation channel TPC1 [12,46], which regulates large-scale wave propagation, can be inhibited by luminal calcium and calcium-induced reactive oxygen species (ROS) [47] from prior activity. Moss contains homologs of these receptors and channels [48] and the decline in moss calcium response over 30 min in these experiments is consistent with the dynamics of these phenomena.

We also demonstrated the novel ability to entrain calcium oscillations to a prescribed frequency of chitin stimulation (Figure 5). As the sudden increase in chitin pulses elicited calcium waves in nearly all ROIs within 1 min, we confirmed that 1 min pulses of chitin could induce singular, synchronized calcium waves across all cells. This external control of calcium wave frequency provides an exciting opportunity to decode how wave frequency modulates gene expression through temporally-sensitive transcription factors [7,11,13]. For example, driving calcium oscillations at different rates and performing RNA-seq could uncover the “frequency map” of the moss genome.

In this study, all cells of the colony were stimulated equally and simultaneously by a uniform, high concentration of chitin oligosaccharides, representing a strong fungal attack [4]. Beyond microfluidic control of chemical *timing*, devices with multiple inlets can control *spatial* chemical patterns by exploiting laminar flow physics [49,50]. Future experiments could stimulate a single cell within the colony by directing a narrow, micron-scale stream of chitin stimulus, and then trace wave propagation from the target cell across the colony. It will be interesting to observe the relation of calcium activity in adjacent unstimulated cells and map wave propagation across cell boundaries under these conditions. This experimental setup may better resemble the realistic initial encounter of a pathogen at a local point on the plant.

The characteristics of calcium waves, including symmetry and consistency, onset delays following stimulus addition, and the inability to halt waves in progress by stimulus removal, all provide hints to the underlying signaling mechanisms. The lack of direct synchronization or calcium propagation across adjacent cells suggests signaling by other molecules. Dynamic mathematical models that produce simulated responses consistent with experimental data will be useful to investigate and identify these signals and to design perturbation experiments to further interrogate calcium signaling [10], for example, by RNA interference [51] and knockout [52] of key regulatory genes. The incorporation of other fluorescent sensors, such as for ROS and voltage changes, further expands the ability to interrogate molecular signaling mechanisms. Together, this toolset should aid in the further study of calcium signaling in plants, including mechanisms of signal propagation, the regulation of gene expression, and protection against external challenge.

## 4. Materials and Methods

### 4.1. Plant Material and Growth Conditions

All plant lines used in this study were derived from the moss *Physcomitrium patens* (Hedw.) Mitt., Gransden strain. Moss lines were cultured in a Percival growth chamber at 25 °C under white light (fluorescent light bulb, cool white GE F17-T8 at ~100 µmol/s/m^2^) and a 16 h/8 h light–dark cycle for 6–8 days before being used in experiments. Sub-culturing was done with an Omni Tissue Homogenizer (TH) set at half power and using 7 mm hard tissue Omni tip plastic probes. Plants were grown on cellophane disks (AA Packaging Limited, Preston, UK) placed on top of 1% agar in moss medium (described below) on 10 cm Petri dishes. One plate of one-week-old tissue was ground in 4 mL of water for ~10 s. Two new plates were inoculated with 1 mL each, and the other two milliliters were sieved with a Falcon disposable cell strainer (70 µm pore size) to select for very small protonemal colonies. The flow-through was placed onto two plates (1 mL each).

### 4.2. Solutions

Solutions were prepared in moss medium, a formulation modified from Knops medium [53], consisting of: 1 mM MgSO_4_·7H_2_O, 1 mM Ca(NO_3_)_2_·4H_2_O, 4 mM KNO3, 89 µM Fe-EDTA, 1.84 mM KH_2_PO_4_, 9.9 µM H_3_BO_3_, 220 nM CuSO_4_·5H_2_O, 1.97 µM MnCl_2_·4H_2_O, 230 nM CoCl_2_·6H_2_O, 190 nM ZnSO_4_·7H_2_O, 168 nM KI, 100 nM NaMoO_4_·2H_2_O. These modifications include an additional 9 µM Fe-EDTA for chelating the Fe-component and phosphate stock solution to adjust pH to 5.5. The reduced pH and lower Ca^2+^ minimized the formation of salt crystals. A 10× stock solution of 22 mg/mL chitin was prepared in water and stored at −20 °C. The chitin stock was thawed and diluted in the media described above to a concentration of 2.2 mg/mL on the day of each experiment. The moss medium was filtered through a 0.45 μm PES syringe filter (Genesee Scientific, El Cajon, CA, USA; Cat#: 25-245) when creating the stimulus solution. Fluorescein (0.1–1 μg/mL) was added to either the stimulus solution or moss medium to verify stimulation flow and timing.

### 4.3. Microfluidic Device Design and Fabrication

To investigate dynamic plant–fungal interactions, we created a monolayer microfluidic device design capable of rapid, reversible switching between chemical stimuli. A chamber height of 35 or 50 μm was chosen to keep small moss protonemal colonies within the 50 μm depth of field of the fluorescent imaging setup. The design features two fluid inlet ports that connect to the imaging area via a T-shaped channel (Figure 1b) to enable the addition, removal, and spatial control of stimulus solutions from the receptive field of the colonies. Devices also feature two U-shaped traps inspired by [22] aligned with each lateral loading port to locate colonies centrally in the imaging area and provide support. The 3 mm diameter imaging area matched the microscope field of view, and the surrounding support posts (100 × 200 μm) prevented collapse when plasma bonding. The device master molds were created by photolithography and soft lithography following methods previously described [26]. Briefly, a 35 μm or 50 μm layer of SU8 2035 photoresist (Kayaku, Westborough, MA USA) was applied to a 4-inch Si wafer (University Wafer, South Boston, MA, USA) and exposed through a 25,000-dpi photomask (Artnet Pro, San Jose, CA, USA) to generate a master mold. Polydimethylsiloxane (PDMS, Sylgard 184, mixed 1:10 by weight) was cast to create ~5 mm thick microfluidic devices. Inflow, loading, and outflow ports were punched using a dermal punch (Miltex, 1 mm), then devices were cleaned and plasma bonded (Harrick PDC-32G, 18 W power for 45 s) to a 25 × 75 mm glass slide. Assembled arenas were degassed in a vacuum desiccator for at least an hour before loading moss medium through the outlet port. This step ensures the removal of air bubbles within the microfluidic channels prior to loading colonies.

### 4.4. Experiment Setup

All solutions were filtered using 0.45 μm PES syringe filters on the day of the experiment. Moss medium and chitin stimulus solutions were placed into 60 mL syringe reservoirs and covered with Parafilm. Microfluidic experiments were prepared by assembling fluid reservoirs and tubing as previously described [26]. Inline 0.45 μm PES filters were connected below each reservoir to prevent aggregates from entering the imaging area. Reservoirs were connected to the microfluidic device using 0.020 “Tygon microbore tubing connected to a blunt 23-gauge Luer stub adapter on one end and a ½” long 19-gauge stainless steel tube on the other end. Bubbles were removed from each fluid line before connecting to the degassed microfluidic device and filling it with moss medium [27]. For each experiment, protonemal colonies were randomly selected from the ground protonemal tissue and placed in a dish of moss medium. Colonies were drawn into loading tubing with a 3 mL syringe, then gently injected into the arena using the loading ports and allowed to adapt to the microfluidic environment for 30 min to 3 h with continuous medium flow before imaging.

### 4.5. Automated Timelapse Imaging and Stimulation

Images were acquired using an inverted widefield epifluorescence microscope (Applied Scientific Instruments, Eugene, OR, USA) with a 4×/0.28 NA objective (Olympus XLFLUOR4X, Tokyo, Japan). Frames were acquired at 1 fps using a Hamamatsu Orca Flash 4.0 sCMOS camera mounted with a 1.0x c-Mount adapter. Binning was set to 2 to enhance the signal-to-noise ratio (SNR) of acquired signals for a spatial resolution of 0.345 pixels/μm (2.9 μm/pixel). A blue LED (Mightex GCS-470-50, North York, ON, Canada) excited GCaMP for 10 ms each acquisition frame. A 3-way solenoid valve (Bio-Chem Fluidics, PN: 075P2-S642, Boonton, NJ, USA) actuated by a ValveLink 8.2 (Automate Scientific, Berkeley, CA, USA) directed a stimulus or medium solution into the microfluidic arena. Image capture, fluorescence illumination, and stimulus delivery were synchronized via digital trigger signals coordinated by an open-source system previously described [26,27]. This system uses Micro-manager microscopy software (version 1.4.24) in streaming mode to acquire timelapse images and communicate experiment parameters by serial commands to an Arduino Uno microcontroller, which coordinates digital trigger pulses.

Timelapse recordings of up to 60 min were conducted at room temperature (20–22 °C), beginning with moss medium flow. Chitin stimulation was introduced after 30 min and persisted for 30 min (Figure 2 and Figure 3), or chitin pulses were repeatedly introduced and removed for different durations and intervals (Figure 4 and Figure 5).

### 4.6. Structural Image Acquisition

After timelapse recordings, microfluidic devices were moved to a Zeiss inverted microscope with an Axiocam 503 camera to identify individual cell borders. Colonies were stained by flowing 0.1 µg/mL Direct Yellow 96 (Solophenyl Flavine 7GFE, US Bio, Salem, MA, USA) through the device for 30 min to label cell wall components. Brightfield and epifluorescence images were acquired at higher magnification (10×/0.3 NA) in Z-stacks with a 1.3 µm step size. Cell borders were manually outlined in FIJI [54] with the segmented line tool and validated between brightfield and stained images. These cell outlines provided the gold standard to evaluate the performance of the cluster generation algorithm and the initial number of ROIs per cell in the clustering algorithm, described below.

### 4.7. Image Processing Pipeline for Automated ROI Segmentation

#### 4.7.1. Foreground–Background Detection

**Figure 6:**
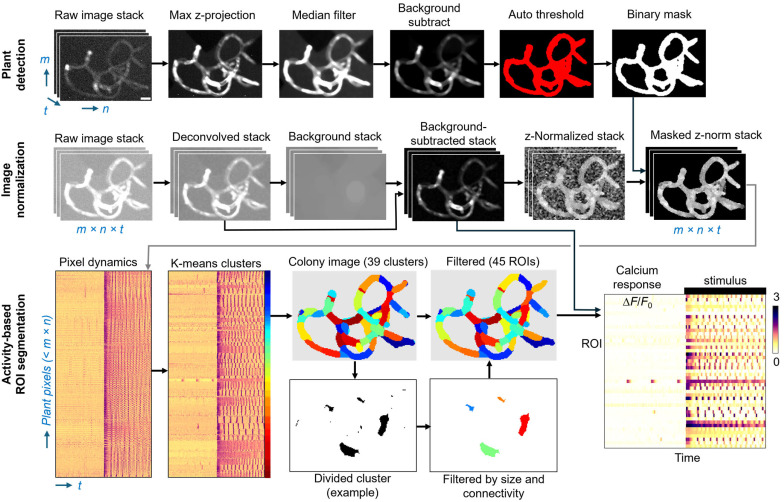
Automated ROI segmentation pipeline and data reduction. (**top row**) A binary mask is generated indicating colony pixels versus background pixels. The raw image stack (3D; *m* × *n* × *t*; in this example, 114 pixels wide × 158 pixels tall × 3600 frames) is reduced to an *m* × *n* binary mask image. (**middle row**) Calcium fluorescence image stacks are normalized to scale-independent Z-scores for each pixel, which equalize all pixels within a cell that respond with similar temporal dynamics. (**bottom row**) Data are flattened to a 2D matrix of each plant pixel Z-score over time (here, 5996 plant pixels × 3600 time points). *K*-means clustering grouped all pixels into 39 predicted clusters based on colony size divided by mean ROI size (150 pixel^2^). Clusters were reorganized to the colony geometry and filtered for size and connectedness. Filtered ROI numbers typically increase over initial cluster number. For example, a divided cluster is shown separated into four ROIs with noise pixels removed. Fluorescence intensity from the background-subtracted stack is integrated across each of the resulting 45 ROIs in this example, normalized to minimum intensity, yielding a reduced 2D dataset (45 ROIs × 3600 timepoints).

Regions of distinct calcium signal dynamics were automatically identified using an image processing pipeline, as depicted in Figure 6. First, raw TIFF stacks generated from the timelapse acquisition were cropped and processed with a FIJI macro. The raw image stack was flattened by maximum z-projection and background subtraction was applied using radius values between 25 and 90 pixels (72–260 µm) depending on colony size. The resulting image was median filtered (radius = 2.5 pixels or 7.2 µm) to reduce noise and maintain contrast around the edges. To identify colony regions, an automatic threshold using the “Triangle” method was applied. Non-colony noise was removed by selecting pixel areas at least 200 pixels^2^ (~1600 µm^2^) and circularity larger than 0.9. Each binary mask was validated by manual inspection.

#### 4.7.2. Fluorescence Image Correction and Normalization

To improve signal focus and reduce background fluorescence, each widefield image stack was deconvolved using a theoretical point spread function (PSF) assuming Abbe resolution limits (0.61 λ/NA = 1.1 µm) using the “deconvolucy” MATLAB function and “edgetaper” to reduce ringing artifacts generated from iterative methods. We set the number of iterations to 10, which consistently generated smoother images and reduced the impact of out-of-focus blur without amplifying noise or introducing artifacts in the images [55]. This deconvolution step improved the outcome of the clustering algorithm, better matching ROIs to plant geometry.

GCaMP fluorescence (*F_pixel_*) was obtained by subtracting background fluorescence from each pixel as follows. The previously generated binary masks were morphologically opened (MATLAB “imopen” function, with “disk” structuring element and radius 15 pixels) to remove any structures smaller than ~10 μm in diameter from the background images. The background images were then subtracted from the deconvolved image stack before quantification.

Fluorescence intensity is greatest in the center of each cell and lowest at the cell borders. Therefore, data were normalized to Z-scores per pixel before ROI detection, to group pixels by their dynamics irrespective of their time-averaged intensity. For each pixel, the mean intensity over time (*µ*) was subtracted from each time point (*x*) and divided by the standard deviation over time (*σ*), i.e., Z=(x−μ)/σ. Z-normalized pixels are more uniform across each cell (Figure 6, middle row left). Next, background pixels were removed using the binary colony mask.

#### 4.7.3. Data Reduction

Contiguous pixel “regions of interest” (ROIs) with similar calcium temporal dynamics were identified by *k*-means clustering on a 2D matrix representation of the Z-normalized image stack. Each row represents a single pixel value over time (Figure 6, lower left). The clustering algorithm groups pixels into a pre-specified number of clusters, estimated initially by the number of cells per colony. Subsequently, the initial number of ROIs was calculated from total colony pixel area divided by the average desired ROI area (150 pixels^2^ or 1260 μm^2^, determined empirically). The initial cluster regions may contain spatially discontinuous regions, which were split into separate ROIs (Figure 6, bottom row). Any small regions below a minimum area (12 pixels or 101 μm^2^) reflected noise and were also removed. As a result, filtered ROIs were relatively insensitive to the initial cluster number. Scaling the initial cluster number from 50% to 150% of the optimal estimate only changed the number of filtered ROIs by 1–3 regions (5–10% variation), reflecting an effective strategy of splitting and pruning ROIs. Clustering used the distance metric “cosine” to maximize the similarity of pixels within the same cluster and the difference between separate clusters. The “OnlinePhase” setting and 10 replicates with random initializations performed best.

Each Ca^2+^ wave was extracted as 120 s windows (60 s before and after the wave peak) and normalized to the calcium peak intensity. Peaks were identified using the “findpeaks” MATLAB function with a prominence threshold of 0.35 times the Median Absolute Deviation, chosen to capture both the spontaneous and chitin-driven oscillations. ROIs with a maximum response less than 100% ΔF/F_0_ were excluded from analysis, as no calcium waves were detected. Due to strong chitin stimulation in all experiments, excluded ROIs were rare (<1%) and included both dim non-responsive regions (non-viable or non-colony) or constitutively bright regions.

### 4.8. Statistical Analysis

Data are represented as the mean ± standard deviation (SD) unless otherwise noted, typically median and interquartile range (25 to 75 percentile) for non-normally distributed distributions. Non-responsive ROIs were excluded from quantification if the maximum increase in fluorescence (Δ*F*) within 5 min of stimulation was less than 100% of the baseline (*F*_0_) fluorescence, as these regions typically lacked calcium responses. In comparison, the typical chitin-induced responses generated initial peaks around 250% Δ*F*/*F*_0_. Sample sizes for each analysis (waves, ROIs, or colonies) are reported in figure legends. Statistics were performed using either a one-way ANOVA with Bonferroni’s correction for multiple comparisons, or two-tailed t-test using the Statistics and Machine Learning Toolbox in MATLAB (v. 2023a).

## Data Availability

Data are available upon request. Analysis scripts and sample data are publicly available at https://github.com/albrechtLab/moss_calcium (accessed on 1 January 2026).

## Supplementary Materials

The following supporting information can be downloaded at: https://www.mdpi.com/article/10.3390/plants15040582/s1. Video S1: Timelapse video of the colony in Figure S1 displayed as pseudocolored background-subtracted fluorescence (left) and ROIs (right), downsampled to show 20 s intervals over one hour, with chitin stimulus added at 1800 s (30 min). Video S2: Timelapse video of the long-adapted, daytime-stimulated protonemal colony in Figure S2, with chitin added at 1800 s (30 min). Video S3: Timelapse video of long-adapted nighttime-stimulated protonemal colony, demonstrating the strong initial blue-light response before chitin addition at 1800 s. Video S4: Timelapse video of a long-adapted, daytime-stimulated protonemal colony from Figure S4, presented five 5-min chitin pulses. Video S5: Timelapse video of a protonemal colony from Figure S5, stimulated with 1-min chitin pulses every 4-min, driving regular colony-wide oscillations. Figure S1: Calcium wave intervals for each ROI and stimulation condition during chitin presentation. Figure S2: Change in calcium wave interval upon chitin stimulation for each ROI and stimulation condition. Figure S3: Stimulus-triggered calcium wave dynamics.

## Abbreviations

The following abbreviations are used in this manuscript:

ACA	Auto-inhibited Ca^2+^-ATPase
ANOVA	Analysis of Variance
AUC	Area Under Curve
CA, CAs	Caulonemal Cell (tip or subapical)
CH, CHs	Chloronemal Cell (tip or subapical)
CV	Coefficient of Variation
CERK1	Chitin Elicitor Receptor Kinase 1
fps	Frame Per Second
FW10	Full Width at 10% Prominence
LED	Light-Emitting Diode
NA	Numerical Aperture
PAMP	Pathogen-Associated Molecular Pattern
PDMS	Poly(dimethyl) siloxane
PES	Polyethersulfone
PSF	Point Spread Function
ROI	Region of Interest
ROS	Reactive Oxygen Species
SD	Standard Deviation
SEM	Standard Error of the Mean
SNR	Signal-to-Noise Ratio
TPC1	Two-Pore Channel 1
