# Supplementary material for: Microfluidic Interrogation of Chitin-Induced Calcium Oscillations in the Moss Physcomitrium patens
Source: Plants (Basel). 2026 Feb 12;15(4):582. doi: 10.3390/plants15040582 (PMC12944216; doi:10.3390/plants15040582)
Supplement: Supplementary file 1 [file plants-15-00582-s001.zip › plants-4083775-Supplementary Figures.pdf]

Supplementary Figures

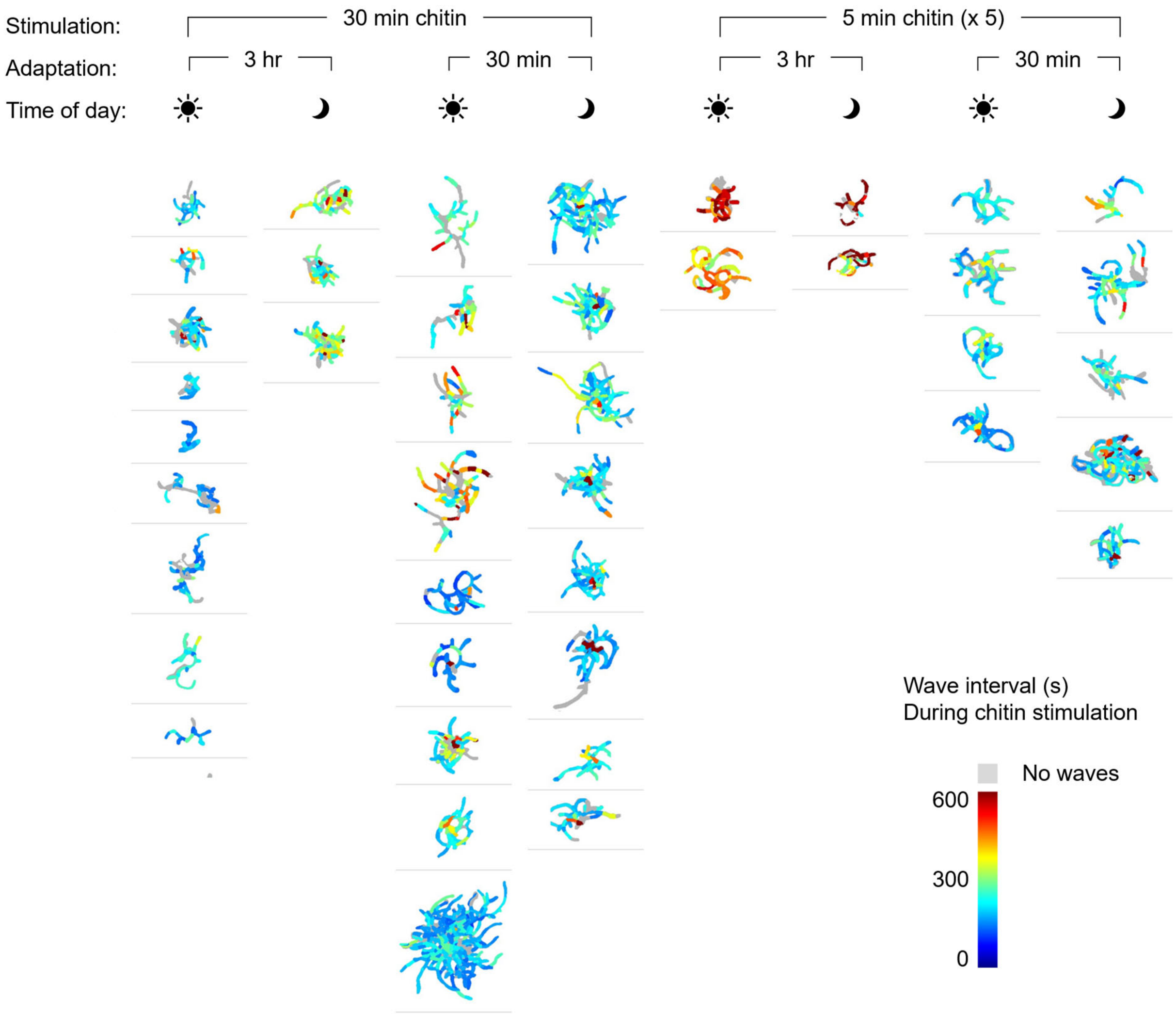

Supplementary Figure S1. Calcium wave intervals for each ROI and stimulation condition during chitin presentation. Each ROI is colored by mean calcium wave interval during a 30 min continuous chitin application (see Fig. 3) or five repeated 5-min presentations (Fig. 4), after 3 hr or 30 min adaptation in the device, and during daytime or night. Gray ROIs did not exhibit calcium waves during stimulation or were not assigned an active ROI.

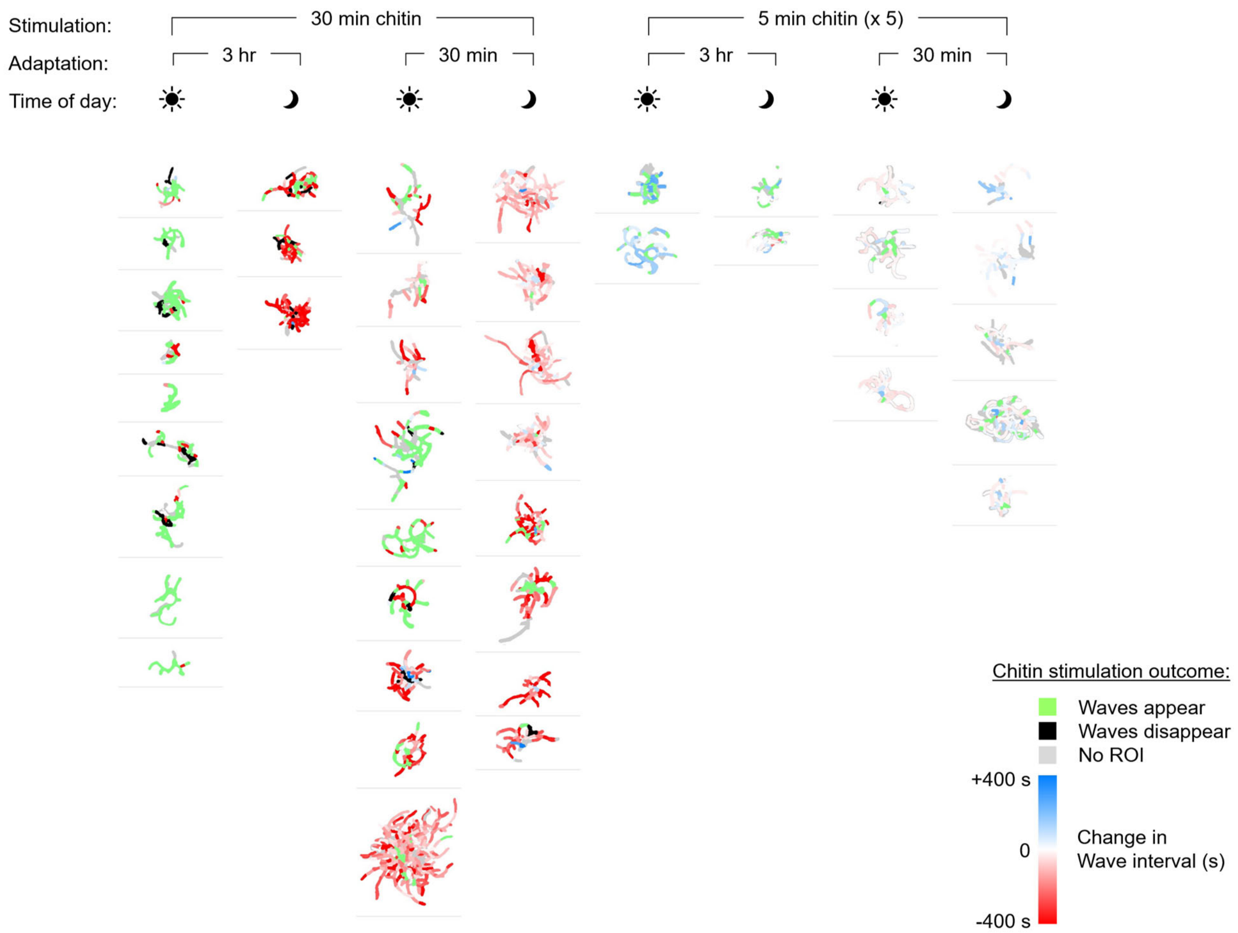

Supplementary Figure S2. Change in calcium wave interval upon chitin stimulation for each ROI and stimulation condition. Green indicates calcium waves appeared during chitin stimulation (i.e., no waves before stimulation); black indicates the loss of calcium waves during chitin stimulation; and grey indicates an ROI that was removed from the analysis.

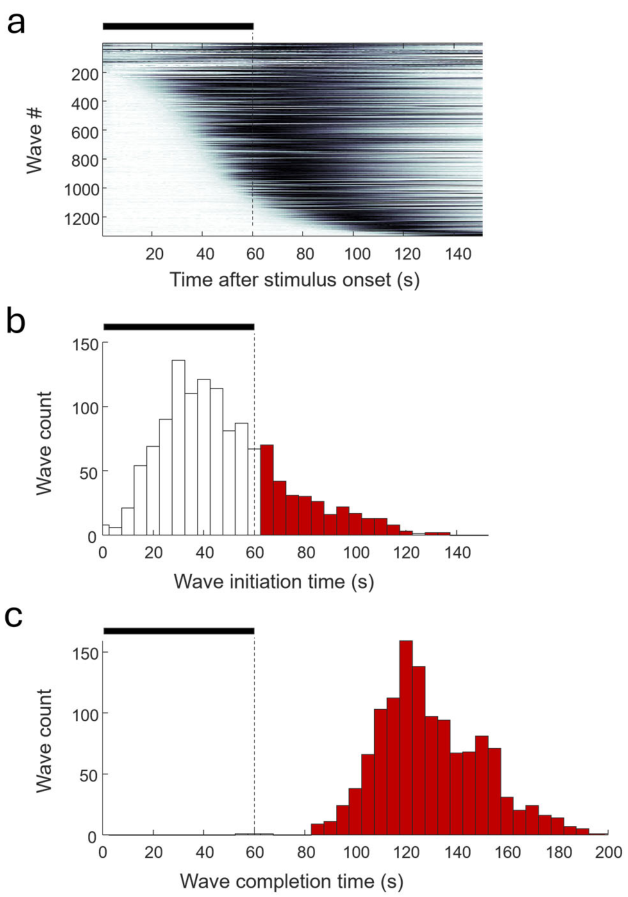

Supplementary Figure S3. Stimulus-triggered calcium wave dynamics. (**a**) Heatmap depicting calcium waves from all ROIs during ten 1-min chitin pulses repeated every 4 min. n = 1260 waves. Shading represents normalized GCaMP fluorescence, where 0 (white) represents the beginning and end of the wave and 1 (black) represents the peak. Bar above indicates chitin pulse timing. Waves are sorted by increasing initiation delay after stimulus onset, defined as the time from chitin addition to calcium rise to 10% of peak fluorescence. (**b**) Histogram of wave initiation delay. Red highlighting indicates waves initiated after removal of the 60-s chitin pulse (26%, 323 of 1260). (**c**) Histogram of wave termination time. All calcium waves concluded well after removal of the chitin pulse (99.8%, 1258 of 1260), with a mean termination delay of 67.8 ± 24.1 s (51.8 - 83.7 s interquartile range).
